# Metformin Inhibits Autophagy, Mitophagy and Antagonizes Doxorubicin-Induced Cardiomyocyte Death

**DOI:** 10.53941/ijddp.0201004

**Published:** 2023-02-17

**Authors:** Jennifer Van, Younghee Hahn, Brett Silverstein, Cairong Li, Fei Cai, Jia Wei, Lokesh Katiki, Puja Mehta, Katherine Livatova, Jaclyn DelPozzo, Tamayo Kobayashi, Yuan Huang, Satoru Kobayashi, Qiangrong Liang

**Affiliations:** 1 Department of Biomedical Sciences, New York Institute of Technology, College of Osteopathic Medicine, Old Westbury, New York 10001, United States; 2 Clinical Medical College, Hubei University of Science and Technology, Xianning 332306, China; 3 Department of Cardiology, the Second Affiliated Hospital, Xi'an Jiaotong University, Xi'an 710000, China

**Keywords:** metformin, autophagy, mitophagy, doxorubicin cardiotoxicity

## Abstract

The antidiabetic drug metformin has been shown to reduce cardiac injury under various pathological conditions, including anticancer drug doxorubicin (DOX)-induced cardiotoxicity, which makes metformin a prime candidate for repurposing. However, the mechanisms that mediate the cardioprotective effects of metformin remain highly controversial. In this study, we tested a prevailing hypothesis that metformin activates autophagy/mitophagy to reduce DOX cardiotoxicity. FVB/N mice and H9C2 cardiac myoblasts were treated with metformin, respectively. Autophagy/mitophagy was determined by Western blot analysis of microtubule-associated protein light chain 3, form-II (LC3-II), a well-established marker of autophagic vesicles. Although metformin had minimal effects on basal LC3-II levels, it significantly inhibited the accumulation of LC3-II levels by the lysosomal protease inhibitors pepstatin A and E64d in both total cell lysates and mitochondrial fractions. Also, dual fluorescent autophagy/mitophagy reporters demonstrated that metformin slowed the degradation rate of autophagic cargos or mitochondrial fragments in the lysosomes. These surprising results suggest that metformin inhibits rather than stimulates autophagy/mitophagy, sharply contrasting the popular belief. In addition, metformin diminished DOX-induced autophagy/mitophagy as well as cardiomyocyte death. Together, these results suggest that the cardioprotective effects of metformin against DOX cardiotoxicity may be mediated by its ability to inhibit autophagy and mitophagy, although the underlying molecular mechanisms remain to be determined.

## 1. Introduction

The anthracycline doxorubicin (DOX) is a widely used and highly effective chemotherapeutic agent for the treatment of a broad spectrum of cancers including various solid tumors and leukemia. However, DOX chemotherapy is associated with severe cardiotoxic effects that culminate in irreversible congestive heart failure [1–3]. Due to the dose-dependent risk, the lifetime cumulative dose of DOX should be limited under 450 mg/m^2^ per patient [1]. A common approach for managing DOX cardiotoxicity is to use a cardioprotective agent during DOX treatment. Antioxidant supplements have shown great efficacy in reducing DOX cardiotoxicity in numerous animal studies [4–6], but results from clinical trials are disappointing [7, 8]. Angiotensin-converting enzyme inhibitors and β-adrenergic receptor blockers are routinely used for treating non-cancer-related heart failure, but they only have marginal beneficial effects on DOX cardiotoxicity which is overshadowed by their adverse effects [9]. Over the years, dexrazoxane has remained the only drug approved by the FDA for reducing DOX cardiotoxicity in humans [10, 11]. However, due to its potential to cause myelosuppression and secondary malignancies, the use of dexrazoxane has been limited to pediatric patients with acute lymphoblastic leukemia and breast cancer patients on high doses of DOX [12–14]. Thus, intense research has been conducted to identify new strategies that can reduce DOX cardiotoxicity but without compromising its anti-tumor activity. In this respect, the antidiabetic drug metformin has been suggested as such a promising dual-function agent that can simultaneously decrease DOX cardiotoxicity [15–19] and increase its anticancer activity [20, 21].

Metformin (MET) is an oral biguanide agent that has been used as the first-line treatment of Type II diabetes for several decades due to its safety, efficacy, and tolerability [22, 23]. The use of MET has led to decreased risk of heart failure [24] and reduced cardiovascular mortality beyond its glycemic control effects [25]. Numerous pre-clinical studies have demonstrated the ability of MET to protect the heart under various disease conditions [26–32], including diabetic cardiomyopathy and DOX-induced cardiotoxicity [15–18]. MET can attenuate radiation cardiotoxicity in breast cancer patients [33], suggesting that MET may also reduce DOX cardiotoxicity in patients receiving DOX chemotherapy. Interestingly, several epidemiological and animal studies have revealed MET’s anti-neoplastic and chemopreventive activities [34, 35], suggesting that MET may be used as an adjunct antitumor agent without the risk of causing cardiac injury. However, large-scale prospective clinical trials in cancer patients are needed to establish MET as a dual-function drug that can reduce DOX cardiotoxicity and at the same time retain or enhance its antitumor activity.

Autophagy is a self-digesting mechanism responsible for the removal of long-lived proteins and damaged organelles by the lysosome. Mitophagy is the process to degrade injured or otherwise dysfunctional mitochondria through the autophagy-lysosome pathway. Mitochondrial injury and disruption are a hallmark feature of DOX cardiotoxicity [36–43]. Although mitophagy is believed to be protective in most cases [44], it can be harmful under certain conditions [45, 46], including DOX-induced cardiotoxicity [47]. MET has been proposed to protect the heart under various conditions by enhancing autophagy and/or mitophagy, based on changes in autophagy markers, including microtubule-associated protein light chain 3, form-II (LC3-II), an integral component of autophagic vesicles [48–51]. When it comes to DOX cardiotoxicity, although a prevailing belief is that metformin reduces DOX cardiotoxicity by enhancing autophagy/mitophagy [15, 18], one study suggests that MET protects the heart by antagonizing DOX-induced autophagy and preserving mitochondrial function [52]. Nevertheless, these studies did not measure autophagy/mitophagy flux, nor did they directly determine the functional role of autophagy/mitophagy in MET-mediated cardioprotection. It remains unclear how MET affects autophagy/mitophagy to reduce DOX cardiotoxicity. In the present study, we determined the effect of MET on autophagy/mitophagy flux and its relationship with MET-mediated reduction in DOX-induced cardiomyocyte death. Our study has generated unexpected results suggesting that MET inhibits rather than stimulates autophagy and mitophagy, which may mediate the ability of MET to diminish DOX-induced cardiomyocyte death, challenging the prevailing hypothesis.

## 2. Materials and Methods

### 2.1. Cell Cultures

The H9c2 cardiac myoblast cells were purchased from America Tissue Type Collection (Manassas, VA; catalog # CRL-1446) and cultured in Dulbecco’s modified essential medium (DMEM, Corning Cellgro, 10–017CV) containing 10% fetal bovine serum, 100 U/mL penicillin and 100 μg/mL streptomycin as described previously [47]. Cells were fed every 2–3 days and used for experiments at 80–90% confluence.

### 2.2. Reagents and Treatments

Metformin (1, 1-Dimethylbiguanide hydrochloride, MW 165.62) was purchased from Sigma and dissolved in distilled water at 20 mg/mL. Mice were administered MET (200 mg per kg body weight) by oral gavage with a feeding needle. MET was used at 1 mM for H9c2 cell culture studies. Doxorubicin (DOX) was from Sigma (D1515). DOX stock solution (1 mM) was made in saline and diluted to a final concentration of 1μM when used in cultured H9c2 cells. Rapamycin (Rap) was purchased from Sigma (R8781), dissolved in dimethyl sulfoxide (DMSO; Sigma, 472301) and used at 20nM in cell culture studies. Pepstatin A (PepA) and E64d were purchased from RPI (P30100, E57050), dissolved in DMSO, and used for measuring autophagy/mitophagy flux. For cultured H9c2 cells, we used PepA at 12.5ng/mL and E64d at 5ng/mL in experiments. For animal studies, we injected mt-Rosella mice intraperitoneally (ip) with PepA and E64d each at 1 mg/kg 4 hours before they were sacrificed. The animal use protocol conformed to the Public Health Service Guide for Care and Use of Laboratory Animals and was approved by the NYITCOM Institutional Animal Care and Use Committee.

### 2.3. Western Blot Analysis

Cardiac tissue and cultured cells were processed for Western blot analysis as described previously [53, 54]. H9c2 cells were washed once in PBS and collected in 1xSDS. Samples were boiled for ten minutes, loaded into a polyacrylamide gel for electrophoresis, and then transferred to polyvinylidene difluoride membranes. After being blocked with 5% milk dissolved in Tris-buffered saline containing 1% Tween 20 for 30 minutes, the blots were incubated with primary and secondary antibodies in 2.5% milk overnight at 4°C. The blots were then washed in Tris-buffered saline for 45 minutes and processed for chemiluminescent detection using Lumigen ECL Ultra (TMA-6 Lumigen, MI, USA), and the images were acquired by using Amersham Imager 600. Protein abundance on Western blots was quantified with ImageJ. The following antibodies were purchased from Cell Signaling (Danvers, MA): microtubule-associated protein light chain 3 (LC3B, #3868), PARP (poly-ADP ribose polymerase, #9542), cleaved Caspase 3 (#9664), β-Actin (#4967), VDAC1 (Voltage-dependent anion channel, #4661), AMP-activated protein kinase α (AMPKα, #5832), Phospho-AMPKα (Thr172, #2535), Acetyl-CoA Carboxylase (ACC, #3662), Phospho-ACC (Ser79, #3661), Beclin 1 (#3738), Atg5 (#2630), Atg7 (#8558), Atg12 (#4180), p62 (#23214), Atg16L1 (#8089), Parkin (# 2132), FUNDC1 (#49240), and α -actinin (#6487). The horseradish peroxidase-conjugated secondary antibodies (sc-2004, sc-2005, sc-2020, and sc-2438) were obtained from Santa Cruz Biotechnology, Inc. (Santa Cruz, CA).

### 2.4. Cell Death Analysis

Cell death with membrane rupture such as necrosis was determined by Propidium iodide (PI) staining, while apoptotic cell death by Western blot analysis of the apoptotic proteins, including cleaved caspase 3 and cleaved PARP, as described previously [47]. Propidium iodide (PI) is a nucleic acid binding fluorescent dye which stains DNA and RNA inside of dead cells, while Hoechst 33342 (Invitrogen) binds to AT-rich regions of the minor grove in DNA and stains nuclei of both live and dead cells. Cells cultured in 96-well plates were incubated with 2μg/mL PI and 1.25μg/mL Hoechst for 10 minutes. The fluorescent images were obtained by using Cytation 5 cell imaging multi-mode reader (Agilent). Two sets of TEXAS Red, DAPI filter and bright field images were captured under a 4x objective lens. Five images were obtained per treatment group. PI-positive cells (stained red) were counted and expressed as a percentage of the total number of cells stained blue by Hoechst. The experiments were repeated at least three times.

### 2.5. Dual fluorescent Autophagy and Mitophagy Reporters

The adenovirus encoding the autophagy reporter mRFP-GFP tandem fluorescent-tagged LC3 (AdtfLC3) was a gift from Dr. Junichi Sadoshima as described [55]. Adenovirus encoding the dual fluorescent mtRosella mitophagy reporter (Admt-Rosella) and transgenic mice expressing mt-Rosella in the heart were described previously [47, 56].

### 2.6. Analysis of Autophagy and Mitophagy

Autophagy/mitophagy activity was determined by Western blot analysis of microtubule-associated protein light chain 3, form-II (LC3-II), a well-established marker of autophagic vesicles. Autophagy/mitophagy was also assessed with Dual fluorescent autophagy and mitophagy reporters. Dual-fluorescent images of H9c2 cells infected with AdtfLC3 or Admt-Rosella were split into red and green channels, and contrast optimized. AdtfLC3 encodes a mRFP-GFP-LC3 fusion protein. The GFP-tagged autophagosome population was quantified using the “green only” channel. The RFP-tagged mature autophagosomes and autolysosomes were quantified using ImageJ’s particle analysis with the size threshold of 0.2 to 50px^2^ to exclude background noise, large aggregates, or nuclei, which were unlikely to represent autophagic foci.

Admt-Rosella expresses a dual-emission biosensor comprising a pH-stable RFP linked to a pH-sensitive GFP, which is targeted to mitochondria with a mitochondrial targeting sequence from human cytochrome C oxidase (COX) subunit VIII. The GFP quenched “red only” mitophagy foci were isolated by subtracting the green channel from the red channel using ImageJ’s Image Calculator after splitting two color channels. After the image optimization, ImageJ’s particle analysis was performed to obtain the number of mitophagy foci with a size threshold of 0.2 to 50px^2^. Similarly, confocal images of cardiac tissue sections from the mt-Rosella mitophagy reporter mice were analyzed by using ImageJ and the mean numbers of red dots or mitophagy events from 3 fields per section were compared between animals from different groups.

At least five images (each containing between 1 and 2 cells) were captured and analyzed per treatment. For autophagy or mitophagy flux analysis, experiments were duplicated with addition of lysosomal protease inhibitors Pepstatin A (12.5ng/mL) and E64D (5ng/mL). Autophagy/mitophagy flux was calculated by subtracting the mean number of puncta without inhibitors from the corresponding mean number of puncta with inhibitors.

### 2.7. Statistical Analysis

Data were presented as the mean ± S.E. One or two-way analysis of variance (ANOVA) was used to analyze the differences between experimental groups followed by Tukey's Multiple Comparison Test GraphPad Prism software. p< 0.05 was considered statistically significant.

## 3. Results

### 3.1. MET Inhibits Autophagy/Mitophagy Flux as Shown by the Protein Levels of LC3-II

**Figure 1.**
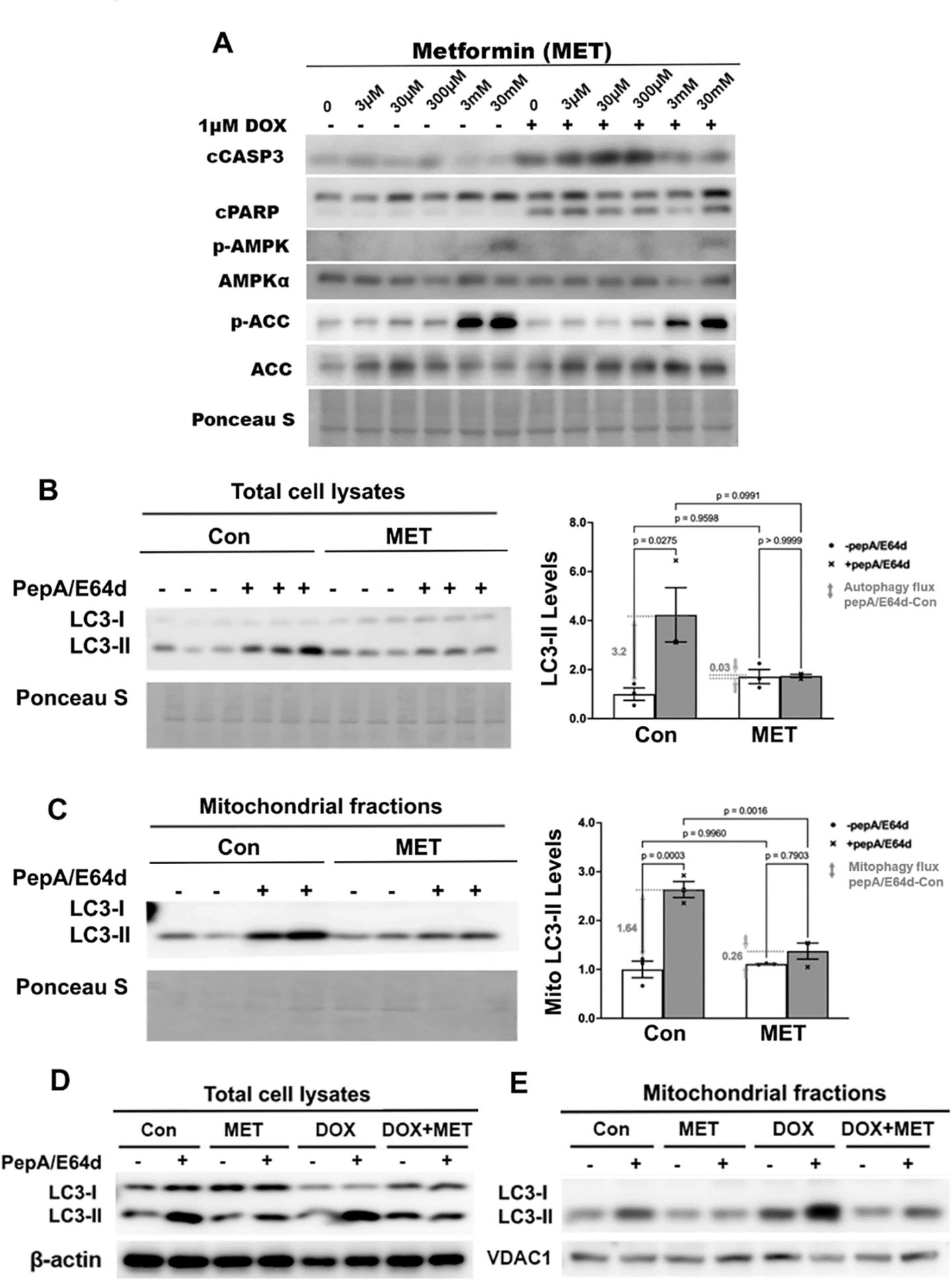
The effects of metformin on the protein levels of LC3-II in H9c2 cells. **A.** H9c2 cardiac blast cells were treated with different doses of MET individually or in combination with 1μM DOX. Protein levels in total cell lysates were determined by Western blot analysis. **B, C, D** and **E**: We pre-treated H9c2 cells with 3 mM MET for 5 hours and then added 1μM DOX and lysosomal inhibitors (PepA and E64d) at the same time. The cells were harvested 16 hours later. The LC3-II levels in total cell lysates or mitochondrial fractions were determined by Western blot analysis followed by quantification using ImageJ. MET abolished the accumulation of LC3-II by PepA and E64d in total cell lysates at baseline **(B)** or in response to DOX **(D)**, and in mitochondrial fractions at baseline **(C)** or in response to DOX **(E)**. Data were expressed as mean ± SD and analyzed by two-way ANOVA (p values were indicated in the bar graphs, n=3 for each treatment).

Previous studies suggest that MET protects the heart through activating autophagy and/or mitophagy, based on the steady state levels of autophagy markers, including microtubule-associated protein light chain 3, form-II (LC3-II), an integral component of autophagic vesicles [48–51]. However, these studies did not determine autophagy/mitophagy flux, which reflects the number of autophagic vehicles that are delivered to and degraded in the lysosome. Autophagy/mitophagy flux can be measured by the difference in the levels of LC3-II protein in the absence and presence of lysosomal inhibitors. We first treated H9c2 cardiac myoblast cells with different doses of MET individually or in combination with 1μM DOX to find the lowest MET dose which can still inhibit DOX-induced cell death. DOX was used at 1μM because this is an optimized dose which consistently induces cardiomyocyte death in numerous studies [15–19, 47, 54, 57–59]. MET did not have any effect on DOX-induced apoptosis until it reached 3 mM dose, as shown by the levels of cleaved caspase 3 (cCASP3) and cPARP (Figure 1A). The 3mM MET also activated AMPK as shown by phosphorylated Acetyl-CoA Carboxylase (p-ACC), an AMPK target. We then determined the effects of 3mM MET on autophagy/mitophagy flux using the lysosomal inhibitors Pepstatin A and E64d. Although MET did not markedly affect the steady state levels of LC3-II in either the total cell lysates (Figure 1B) or mitochondrial fractions (Figure 1C), it blocked the accumulation of LC3-II caused by the lysosomal inhibitors, indicating that MET decelerated autophagic flux (Figure 1B) and mitophagic flux (Figure 1C). These surprising results suggest that MET inhibits rather than stimulates autophagy and mitophagy, sharply contrasting the popular belief. In addition, MET inhibited DOX-induced autophagy (Figure 1D) and mitophagy (Figure 1E) in H9c2 cells as shown by the altered LC3-II levels in total cell lysates (Figure 1D) and mitochondrial fractions (Figure 1E). Together, our results demonstrated the ability of MET to inhibit autophagy and mitophagy at baseline and in response to DOX treatment, suggesting the possibility that MET may reduce DOX cardiotoxicity through inhibiting autophagy and mitophagy.

### 3.2. MET Inhibits Autophagy as Shown by the Dual Fluorescent Autophagy Reporter tfLC3

**Figure 2.**
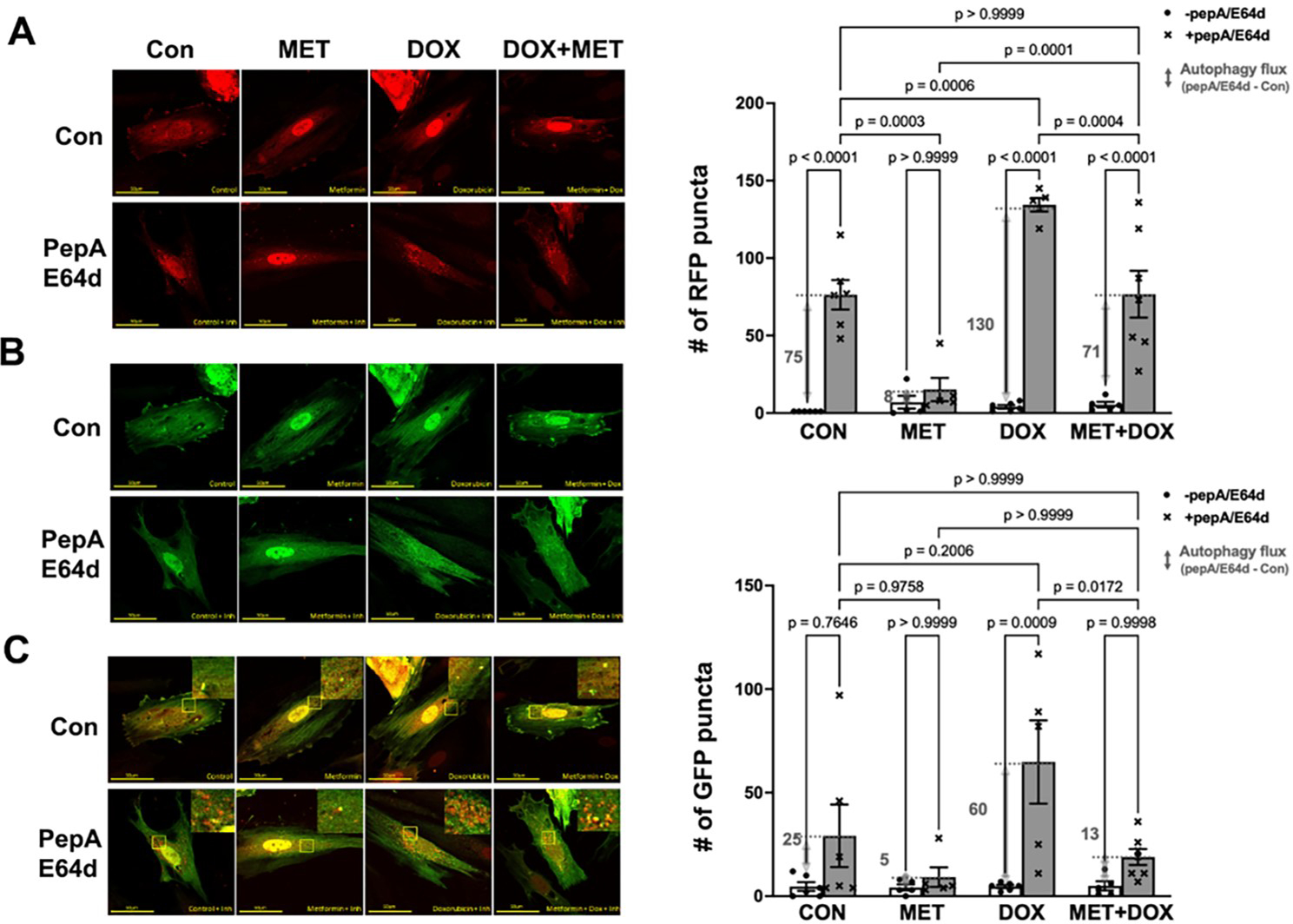
Metformin inhibits autophagy flux in H9c2 cells as shown by the autophagy reporter tfLC3. H9c2 cells were infected with an adenovirus expressing mRFP-GFP tandem fluorescent-tagged LC3 (tfLC3) and treated with 3 mM MET for 5 hours and followed by 1μM DOX either with or without the lysosomal protease inhibitors PepA and E64d. Confocal images were obtained 16 hours later and analyzed with ImageJ. **A.** Confocal images from red channel: the RFP puncta represent both autophagosomes and autolysosomes; **B.** Confocal images from green channel: the GFP puncta are indicative of autophagosomes; and **C.** Merged confocal images: yellow puncta are colocalized GFP and RFP, which indicate autophagosomes, whereas the free RFP signal that does not overlay with the GFP in the merged image is indicative of autolysosomes. The numbers of fluorescent puncta (GFP or RFP dots) were quantified using ImageJ’s particle analysis with the size threshold of 0.2 to 50 px^2^ to exclude background noise, large aggregates, or nuclei, which were unlikely to represent autophagic foci. The scale bars represent 50 μm. Data in the bar graphs are means ± SD and analyzed by one-way ANOVA (p values were indicated in the bar graphs, n=5–7).

To confirm the inhibitory effects of MET on autophagy as suggested by the LC3-II results, we used the dual fluorescent autophagy reporter tfLC3 in which the LC3 was tagged with tandem monomeric RFP (mRFP) and GFP. RFP but not GFP can still produce fluorescence in the acidic environment of lysosomes. Therefore, the GFP puncta are indicative of autophagosomes while the RFP puncta represent both autophagosomes and autolysosomes. As shown in Figure 2A, cells in control group showed a few red puncta, the number of which was markedly increased by the lysosomal inhibitors, indicating an active autophagy flux at baseline. However, MET treatment led to dramatic reductions in the number of red puncta either with or without PepA/E64d, suggesting that MET decelerated autophagy flux at baseline. In addition, DOX accelerated autophagy flux as shown by a significant accumulation of red puncta by PepA/E64d, which was attenuated by MET. The effects of MET on red puncta were also observed on green puncta in Figure 2B, confirming the ability of MET to inhibit autophagy either with or without DOX treatment.

### 3.3. MET Inhibits Mitophagy as Shown by the Dual Fluorescent Mitophagy Reporter Mt-Rosella

**Figure 3.**
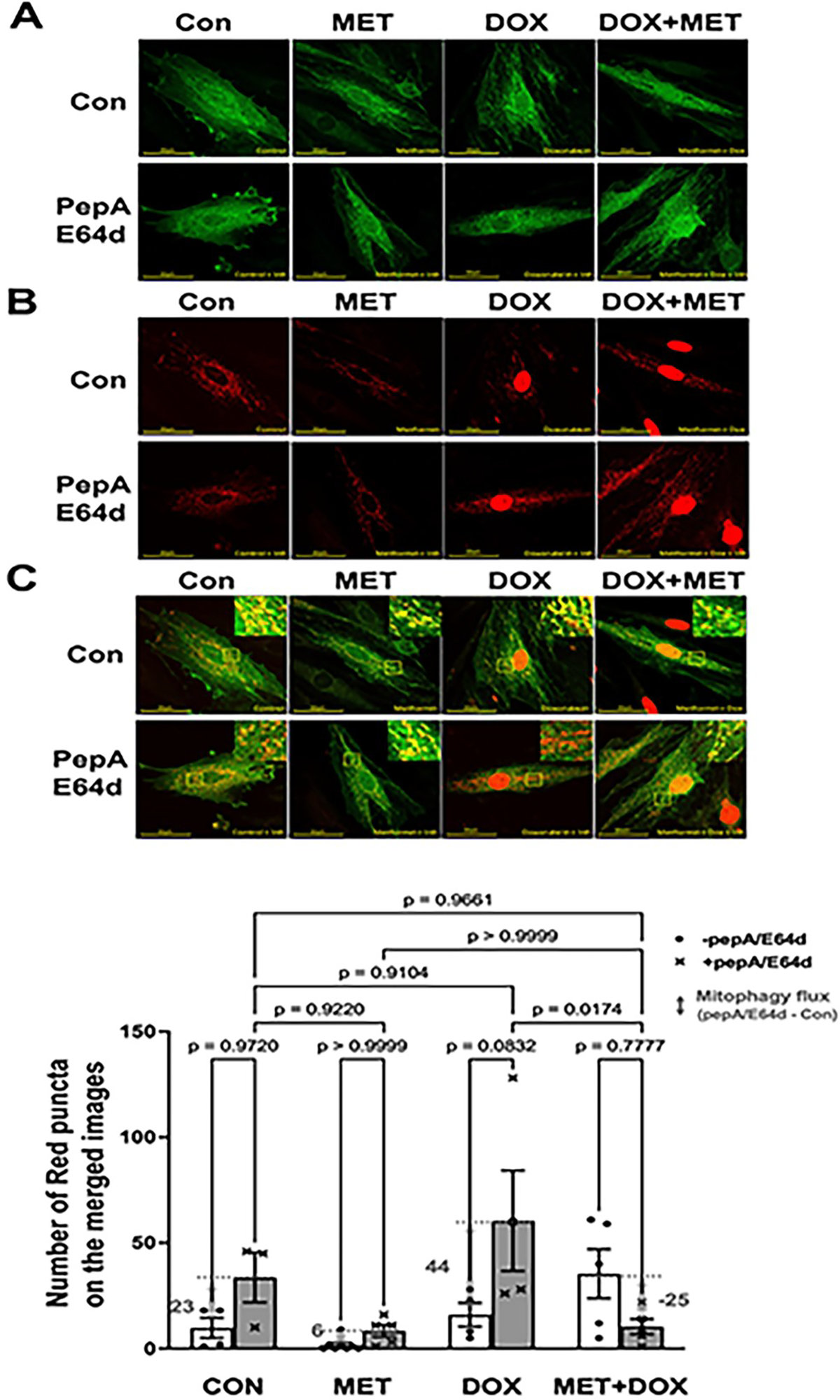
Metformin inhibits mitophagy flux in H9c2 cells as determined by the Rosella mitophagy reporter. H9c2 cells were infected with the Admt-Rosella and treated with 3 mM MET for 5 hours and followed by 1 μM DOX either with or without the lysosomal protease inhibitors PepA and E64d. Confocal images were obtained 16 hours later and analyzed with ImageJ. **A.** Confocal images from green channel: the GFP puncta are indicative of mitophagosomes; **B.** Confocal images from red channel: the RFP puncta represent both mitophagosomes and mitolysosomes, and **C.** Merged confocal images: The red dots or puncta on the overlaid images represent mitophagy events or foci, indicating fragmented mitochondria that are trapped and being degraded in the lysosome. The numbers of mitophagy foci were quantified using ImageJ’s particle analysis with the size threshold of 0.2 to 50 px^2^ to exclude background noise, large aggregates, or nuclei, which were unlikely to represent mitophagic foci. At least five images (each containing between 1 and 2 cells) were captured and analyzed per treatment. The scale bars represent 50 μm. Data in the bar graphs are means ± SD and analyzed by two-way ANOVA (p values were indicated in the bar graphs, n=5–7).

We infected H9c2 cardiac myoblasts with Ad-mt-Rosella which encodes a novel mitophagy reporter. This reporter can faithfully label and track mitochondrial fragments which are sequestered by the autophagosome, and delivered to and degraded in the lysosome [56]. As shown in the merged confocal images (Figure 3C), the mitochondria glowing green or yellow are in the cytosolic compartments, while the fragmented mitochondria fluorescing only red (red puncta) are being degraded within the lysosomes where the pH is low and the GFP is quenched. PepA/E64d led to a marked increase in the number of red dots or puncta in cells treated with DOX, which was significantly reduced by MET, indicating that MET slowed DOX-induced mitochondrial degradation or mitophagy.

### 3.4. Metformin Antagonizes DOX-induced Cardiomyocyte Death

**Figure 4.**
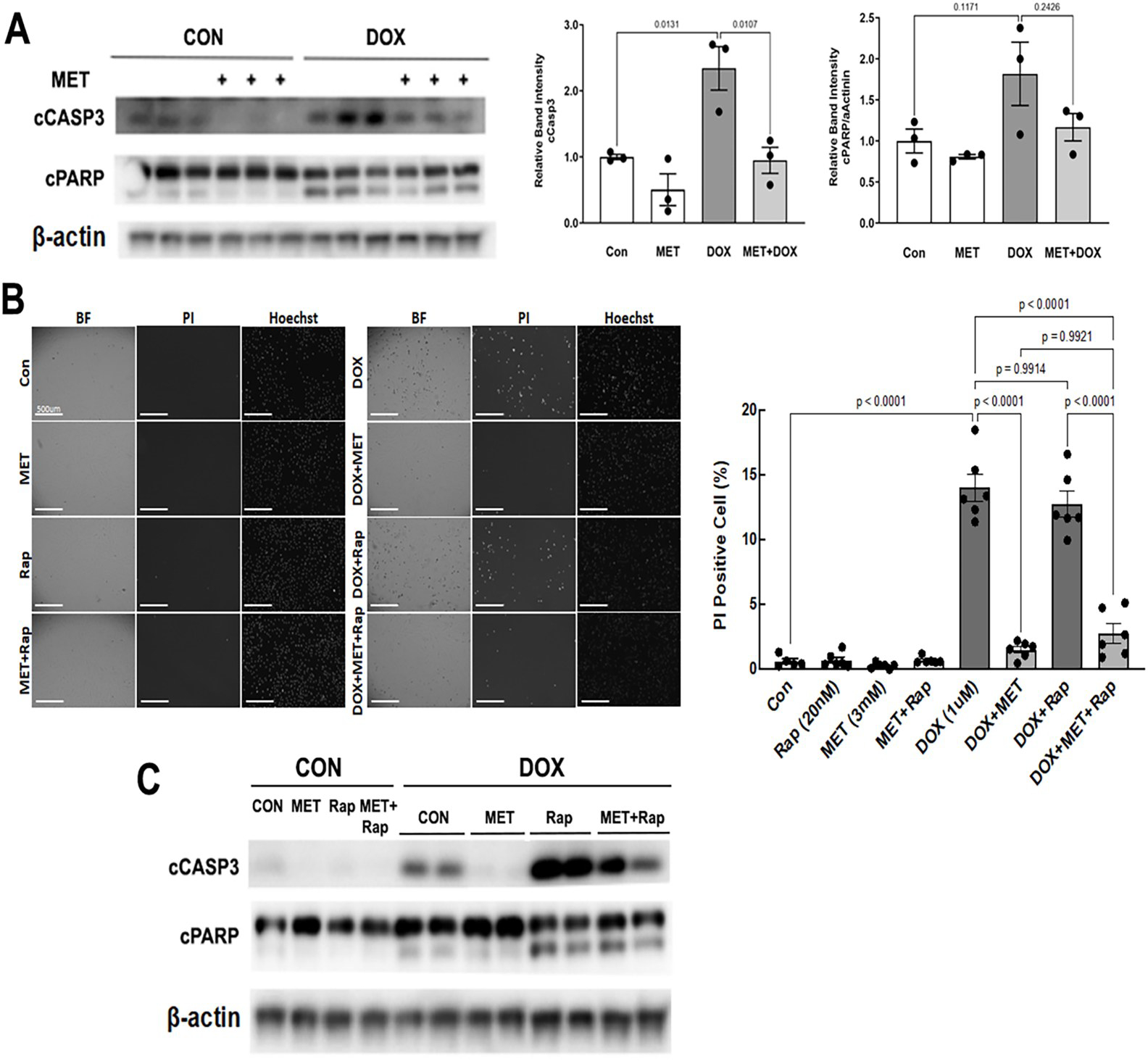
MET diminished DOX-induced cell death. H9c2 cardiac myoblast cells were treated with 3 mM MET for 5 hours and followed by 1μM DOX for 16 hours. **A.** Western blot analysis of cleaved caspase 3 (cCASP3) and PARP (cPARP) followed by quantification using ImageJ. Data were expressed as mean ± SD and analyzed by two-way ANOVA (p values were indicated in the bar graphs, n=3). **B.** Determination of PI positive cells: Cells cultured in 96-well plates were incubated with 2μg/mL PI and 1.25μg/mL Hoechst for 10 minutes. The fluorescent images were obtained by using Cytation 5 cell imaging multi-mode reader (Agilent). Two sets of TEXAS Red, DAPI filter and bright field images were captured under a 4x objective lens. Five images were obtained per treatment group. PI-positive cells (stained red) were counted and expressed as a percentage of the total number of cells stained blue by Hoechst. The scale bars represent 500 μm. Data were expressed as mean ± SD and analyzed by two-way ANOVA (p values were indicated in the bar graphs, n=6). **C.** H9c2 cardiac myoblast cells were treated with 3mM MET, 20nM Rapamycin (Rap), and 1μM DOX, individually or in combination. The cCASP3 and cPARP were evaluated with Western blot analysis.

We determined DOX-induced cardiomyocyte death (Figure 4) by PI staining, which estimates the number of dead cells regardless of the cause of death. Apoptosis was determined by cleaved caspase 3 and PARP. As expected, DOX treatment led to increased cleavage of caspase 3 (Figure 4A) and PI-positive cells (Figure 4B), demonstrating the sufficiency of DOX to induce both necrosis and apoptosis in cardiomyocytes. However, DOX-induced cell death was markedly inhibited by MET. Of note, MET also diminished cell death in the presence of both Rapamycin (Rap) and DOX as shown by decreased PI positive cells (Figure 4B) and reduced cleavage of caspase 3 (Figure 4C). These results suggest that MET can antagonize DOX-induced cardiomyocyte death, which may be mediated by the inhibition of autophagy.

### 3.5. MET Inhibited Mitophagy in the Mouse Heart as Shown by the Mt-Rosella Mitophagy Reporter

**Figure 5.**
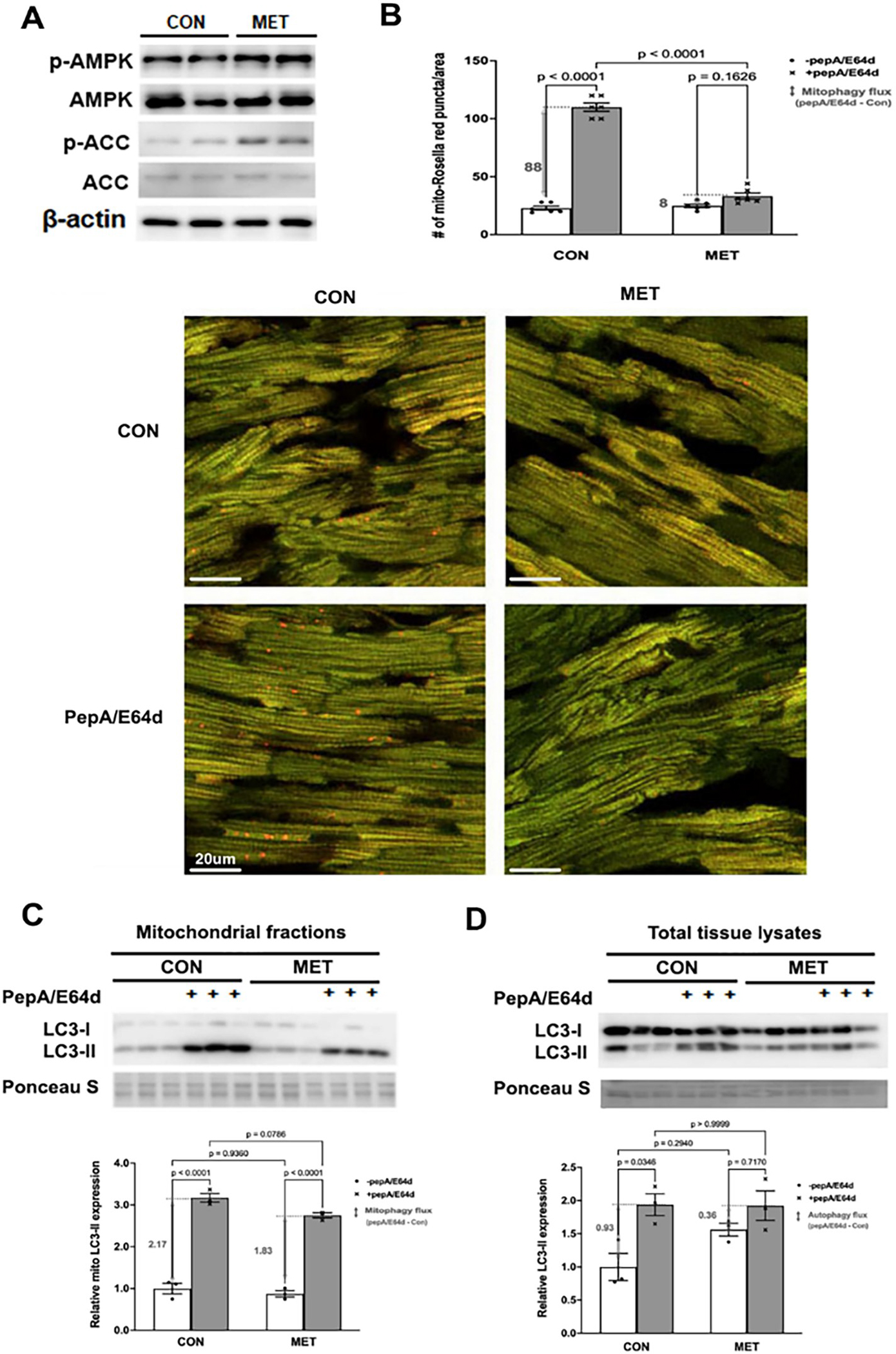
MET inhibited mitophagy in the mouse heart as shown by the mt-Rosella mitophagy reporter. Three-month old mt-Rosella mice received MET (200 mg/kg, oral) or vehicle for 2 days and were injected ip with PepA/E64d (1 mg/kg) 4 hours before mice were sacrificed. **A.** Western blot analysis of AMPK and its effector ACC in cardiac tissue lysates. **B.** Merged Confocal images from cardiac tissue sections were analyzed by using ImageJ and the mean numbers of red dots or mitophagy events from 3 fields per section were compared. The scale bars represent 20 μm. Data were expressed as mean ± SD and were analyzed by one-way ANOVA (p values were indicated in the bar graphs, n=5–6). **C.** Western blot analysis of LC3-II in cardiac mitochondrial fractions. **D.** Western blot analysis of LC3-II in cardiac tissue lysates. Data were expressed as mean ± SD and were analyzed by one-way ANOVA (p values were indicated in the bar graphs, n=3).

We determined the ability of MET to affect mitophagy in vivo using the novel mt-Rosella mitophagy reporter as described [56]. Three-month old mt-Rosella mice received MET (200 mg/kg, oral) or vehicle for 2 days and were injected ip with PepA/E64d (1mg/kg) 4 hours before mice were sacrificed. MET was sufficient to activate cardiac AMPK, a putative positive regulator of autophagy, as shown by increased levels of phosphorylated AMPKα and ACC (Figure 5A). The number of red dots in control heart was notably increased by PepA/E64d (Figure 5B), showing an active baseline mitophagy. However, MET reduced the number of red dots either with or without PepA/E64d, suggesting that MET inhibited mitophagy flux at baseline. This result was confirmed by the amounts of LC3-II in the mitochondrial fractions (Figure 5C). Also, there was a tendency for MET to reduce autophagy flux as shown by the LC3-II levels in the total cardiac tissue lysates (Figure 5D). In summary, MET activated AMPK but paradoxically inhibited the recruitment of autophagy machinery to the mitochondria and decelerated mitophagy flux in the heart, in contrast to the prevailing notion that MET activates mitophagy.

### 3.6. MET Did Not Affect the Protein Expression Levels of Major Autophagy-Related Genes

**Figure 6.**
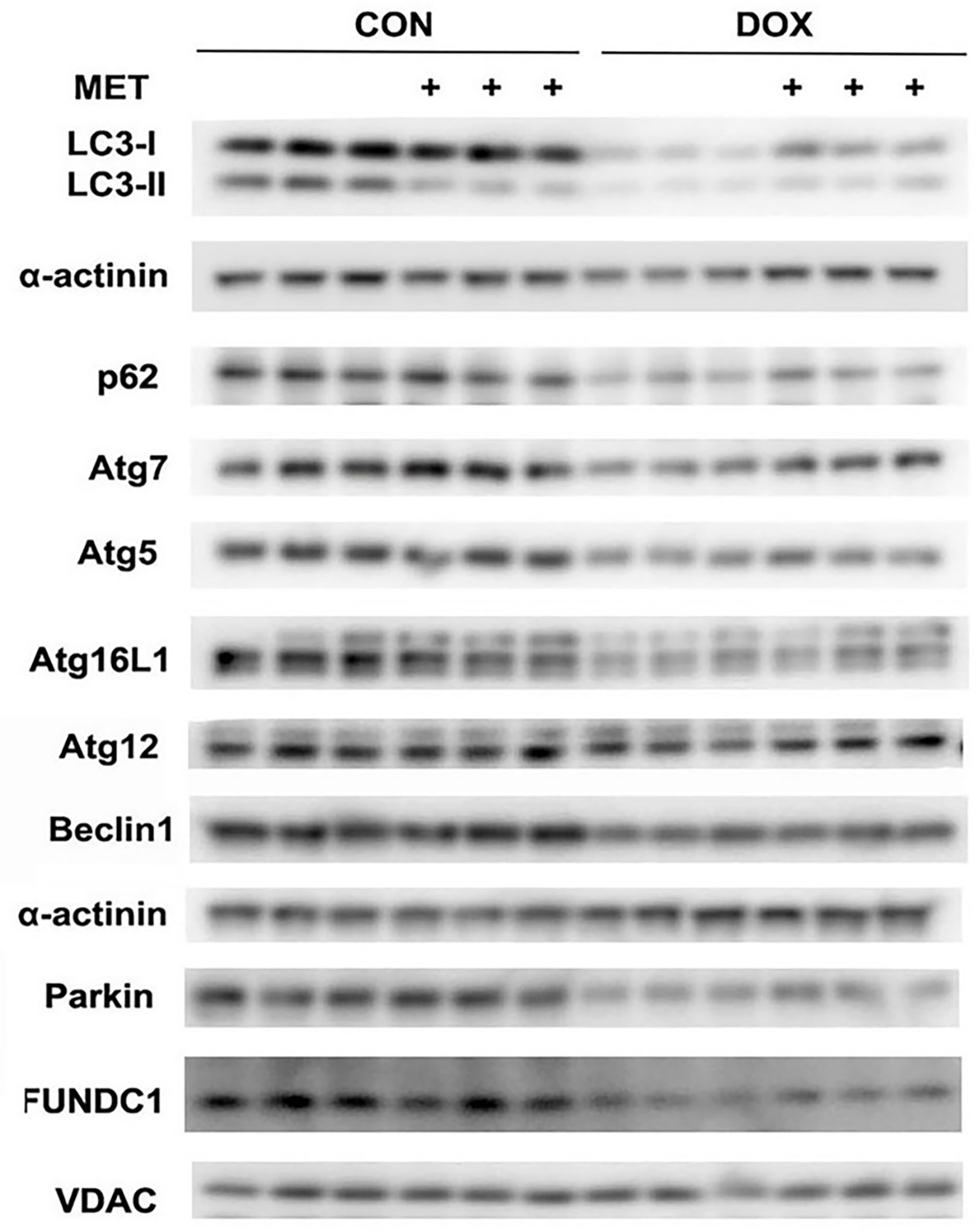
MET did not affect the protein expression levels of major autophagy-related genes. H9c2 cardiac myoblast cells were treated with 3 mM MET and 1μM DOX, individually or in combination. The protein expression levels of autophagy-related genes were determined by Western blot analysis, including Atg5, Atg7, Atg12, Beclin1, Atg16L1, p62, Parkin and FUNDC1.

To explore the mechanisms by which MET inhibits autophagy/mitophagy flux, we determined the protein expression levels of some common autophagy-related genes, including Atg5, Atg7, Atg12, Beclin1, Atg16L1, p62, Parkin and FUNDC1 (FUN14 domain containing 1). Parkin and FUNDC1 are two pathways that positively regulate mitophagy. Surprisingly, despite its ability to inhibit autophagy/mitophagy, MET did not affect the protein expression levels of these autophagy-related genes, either in the absence or presence of DOX (Figure 6).

## 4. Discussion

Although MET is a classical antidiabetic drug widely used for treating type 2 diabetes, numerous studies have demonstrated the ability of MET to exert pleiotropic effects on the body, which have ignited enormous enthusiasm for re-purposing MET for clinical uses other than anti-diabetes, such as anticancer, antiaging, and cardioprotection [26–32]. Indeed, MET has been shown to reduce DOX cardiotoxicity [15–18] and to enhance the anti-cancer activity of DOX in numerous animal studies [18, 20, 21]. Also, clinical trials have shown that MET induces favorable cellular and molecular changes in cancer patients [60–63]. It is thus reasonable to believe that MET is a dual-function drug that can reduce DOX cardiotoxicity without compromising the antitumor activity of DOX. Several hypotheses have been proposed to explain the cardioprotective effects of MET on DOX cardiotoxicity, including reduction of oxidative stress, improvement of mitochondrial function, activation of AMP-activated protein kinase (AMPK) [18], and modulation of autophagy/mitophagy [15].

Autophagy is an intracellular degradation pathway that escorts cytoplasmic cargoes to the lysosome where the cargoes get decomposed and recycled. Mitophagy is the process in which injured or otherwise dysfunctional mitochondria are degraded through the autophagy-lysosome pathway. Autophagy/mitophagy can be either protective or detrimental to the heart depending on the specific context. The exact function of autophagy or mitophagy in DOX cardiotoxicity remains controversial [19], probably due to the differences in the experimental models and the dose and duration of DOX treatment [64, 65]. Indeed, DOX has been shown to either activate autophagy [54, 58, 59, 65, 66] or inhibit autophagy [67–69], but paradoxically, both of which contribute to cardiotoxicity. The same paradox exists when it comes to the effect of DOX on mitophagy and the functional role of mitophagy in DOX cardiotoxicity [47, 70–72]. The controversy may partially result from the methods used to determine mitophagy. The true activity of mitophagy should not be determined solely by a snapshot of the steady state levels of mitophagy markers such as LC3-II but by mitophagy flux. The latter reflects the number of mitochondrial fragments that are delivered to and degraded in the lysosome, which can be measured by the difference in the levels of LC3-II protein associated with mitochondria in the absence and presence of lysosomal inhibitors. Using LC3-II and a novel mt-Rosella mitophagy reporter, we previously showed that DOX accelerated mitophagy flux, which contributed to cardiomyocyte death [47]. The mt-Rosella mitophagy reporter is advantageous for determining mitophagy flux as opposed to mito-Keima [73] which is resistant to acid proteases and may not be efficiently degraded in the lysosome. In addition, the mt-Rosella is in the mitochondrial matrix and may more faithfully track the whole process of mitochondrial degradation as opposed to the mito-QC [74] which is targeted to the outer mitochondrial membrane and may lose track of mitochondrial fragments after outer membrane has been degraded. Although mitochondria-associated LC3-II is the most widely used marker for determining mitophagy flux and mt-Rosella is an excellent mitophagy reporter, it is still valuable to measure the levels of proteins from different mitochondrial subcompartments, mitochondrial DNA content and citrate synthase activity. Collectively, these measurements can provide more comprehensive information regarding the dynamic process of mitochondrial degradation [75]. The outer mitochondrial membrane (OMM) proteins such as mitofusins, TOM complex proteins such as TOM20, and voltage-dependent anion channel (VDAC), can be degraded by the ubiquitin proteasome system. Thus, the loss of these proteins may not necessarily indicate the occurrence of mitophagy. By comparison, the mt-Rosella reporter can monitor all steps of the mitophagic process, generating definitive evidence for mitochondrial degradation as we described previously [56]

MET has been proposed to protect the heart under various conditions by enhancing autophagy and/or mitophagy, based on changes in autophagy markers, including LC3-II, an integral component of autophagic vesicles [48–51]. When it comes to the protective effects of MET on DOX cardiotoxicity, a dominant view is that MET reduces DOX cardiotoxicity by enhancing autophagy/mitophagy [15, 18]. However, an opposite notion suggests that MET protects the heart by antagonizing DOX-induced autophagy and preserving mitochondrial function [52]. To clarify this controversy, we determined the effects of MET on autophagy/mitophagy flux in H9c2 cells and in mouse heart. We found that MET significantly inhibited the autophagy/mitophagy flux at baseline and in response to DOX treatment, as shown by a reduced accumulation of LC3-II levels by the lysosomal protease inhibitors pepstatin A and E64d in both total cell lysates and mitochondrial fractions. This was further confirmed by dual fluorescent reporters for autophagy and mitophagy showing that MET slowed the degradation rate of autophagic cargos or mitochondrial fragments in the lysosomes. These surprising results suggest that MET inhibits rather than stimulates autophagy and mitophagy, in sharp contrast to the prevailing belief. In addition, the inhibition of autophagy/mitophagy by MET was associated with diminished cardiomyocyte death induced by DOX. Notably, MET also attenuated DOX-induced cell death in the presence of rapamycin (Rap) as shown by decreased PI positive cells (Figure 4B) and reduced cleavage of caspase 3 (Figure 4C). Rap is a well-established inhibitor of mTOR kinase pathway and an inducer of autophagy which exacerbated DOX-induced cell death (Figure 4B, 4C), consistent with previous studies [58]. These results suggest that MET can antagonize DOX-induced cardiomyocyte death and this effect is likely mediated by the inhibition of autophagy/mitophagy. As we showed previously [47] and in the present study, DOX causes mitochondrial depolarization and triggers excessive degradation of mitochondria, which disrupt ATP production, contributing to DOX cardiotoxicity. Thus, metformin inhibition of DOX-induced mitophagy could preserve the number of mitochondria and maintain the production of ATP, promoting cardiomyocyte survival. Of course, the dose of MET needs to be titrated to avoid excessive inhibition of mitophagy and thus accumulation of damaged mitochondria. Indeed, the benefit of MET is somewhat lost when it was used at 30 mM as shown in Figure 1A.

## 5. Conclusion

In summary, MET has been safely used to treat diabetes for several decades and has shown pleiotropic effects beyond glucose-lowering, which makes it a prime candidate for repurposing. The protective role of MET in DOX cardiotoxicity has been confirmed in numerous animal studies. However, the underlying mechanisms remain unclear. In the present study, we determined the effect of MET on autophagy/mitophagy flux and its relationship with MET-mediated reduction in DOX-induced cardiomyocyte death. Our study has generated surprising results demonstrating the ability of MET to inhibit rather than stimulate autophagy/mitophagy, which may be one of the mechanisms by which MET attenuates DOX cardiotoxicity. Unexpectedly, despite its ability to inhibit autophagy/mitophagy, MET did not affect the protein expression levels of major autophagy-related genes, either in the absence or presence of DOX (Figure 6). Thus, it remains unclear how metformin can negatively regulate autophagy/mitophagy. Studies are underway to understand the molecular mechanisms that mediate the inhibitory effects of MET on autophagy/mitophagy. Also, further in vivo studies using tumor-bearing animal models and prospective clinical trials are needed to establish MET as an effective adjuvant agent to reduce DOX cardiotoxicity without compromising the antitumor efficacy of DOX.

## Abbreviations:

DOX: doxorubicin
MET: metformin
LC3: microtubule-associated protein light chain
cCasp3: cleaved Caspase3
PARP: Poly (ADP-ribose) polymerase
AMPK: AMP-activated protein kinase
ACC: Acetyl-CoA Carboxylase
FUNDC1: FUN14 domain containing 1
DMEM: Dulbecco’s modified essential medium
DMSO: dimethyl sulfoxide
GFP: green fluorescence protein
mRFP: monomeric red fluorescence protein
PepA: Pepstatin A
PI: Propidium iodide
